# Elucidating treatment targets and mediators within a confirmatory efficacy trial: study protocol for a randomized controlled trial of cognitive-behavioral therapy vs. light therapy for winter depression

**DOI:** 10.1186/s13063-022-06330-9

**Published:** 2022-05-12

**Authors:** Kelly J. Rohan, Peter L. Franzen, Kathryn A. Roeckelin, Greg J. Siegle, David J. Kolko, Teodor T. Postolache, Pamela M. Vacek

**Affiliations:** 1 Department of Psychological Science, University of Vermont, 2 Colchester Avenue, Burlington, VT 05405-0134 USA; 2 Department of Psychiatry, University of Pittsburgh, Thomas Detre Hall, 3811 O’Hara Street, Pittsburgh, PA 15213 USA; 3 Department of Psychology, University of Pittsburgh, 4110 Sennott Square, 210 S Bouquet Street, Pittsburgh, PA 15260 USA; 4 University of Maryland School of Medicine, 655 West Baltimore Street, Baltimore, MD 21201-1559 USA; 5 Biomedical Statistics Research Core, University of Vermont Larner College of Medicine, 25 Hills Building, 111 Colchester Avenue, Burlington, VT 05401-0134 USA

**Keywords:** Seasonal affective disorder, Cognitive-behavioral therapy, Light therapy, Randomized clinical trial, Mediation, Experimental therapeutics, Depression recurrence, Biomarkers

## Abstract

**Background:**

This study is a confirmatory efficacy trial of two treatments for winter seasonal affective disorder (SAD): SAD-tailored group cognitive-behavioral therapy (CBT-SAD) and light therapy (LT). In our previous efficacy trial, post-treatment outcomes for CBT-SAD and LT were very similar, but CBT-SAD was associated with fewer depression recurrences two winters later than LT (27.3% in CBT-SAD vs. 45.6% in LT). CBT-SAD engaged and altered a specific mechanism of action, seasonal beliefs, which mediated CBT-SAD’s acute antidepressant effects and CBT-SAD’s enduring benefit over LT. Seasonal beliefs are theoretically distinct from LT’s assumed target and mechanism: correction of circadian phase. This study applies the experimental therapeutics approach to determine how each treatment works when it is effective and to identify the best candidates for each. Biomarkers of LT’s target and effect include circadian phase angle difference and the post-illumination pupil response. Biomarkers of CBT-SAD’s target and effect include decreased pupillary and sustained frontal gamma-band EEG responses to seasonal words, which are hypothesized as biomarkers of seasonal beliefs, reflecting less engagement with seasonal stimuli following CBT-SAD. In addition to determining change mechanisms, this study tests the efficacy of a “switch” decision rule upon recurrence to inform clinical decision-making in practice.

**Methods:**

Adults with SAD (target *N* = 160) will be randomzied to 6-weeks of CBT-SAD or LT in winter 1; followed in winter 2; and, if a depression recurrence occurs, offered cross-over into the alternate treatment (i.e., switch from LT➔CBT-SAD or CBT-SAD➔LT). All subjects will be followed in winter 3. Biomarker assessments occur at pre-, mid-, and post-treatment in winter 1, at winter 2 follow-up (and again at mid-/post-treatment for those crossed-over), and at winter 3 follow-up. Primary efficacy analyses will test superiority of CBT-SAD over LT on depression recurrence status (the primary outcome). Mediation analyses will use parallel process latent growth curve modeling.

**Discussion:**

Consistent with the National Institute of Mental Health’s priorities for demonstrating target engagement at the level of Research Domain Criteria-relevant biomarkers, this work aims to confirm the targets and mechanisms of LT and CBT-SAD to maximize the impact of future dissemination efforts.

**Trial registration:**

ClinicalTrials.gov identifier: NCT03691792. Registered on October 2, 2018.

## Administrative information

Note: the numbers in curly brackets in this protocol refer to SPIRIT checklist item numbers. The order of the items has been modified to group similar items (see http://www.equator-network.org/reporting-guidelines/spirit-2013-statement-defining-standard-protocol-items-for-clinical-trials/).

**Table:** 

Title {1}	Elucidating Treatment Targets and Mediators within a Confirmatory Efficacy Trial: Study Protocol for a Randomized Controlled Trial of Cognitive-Behavioral Therapy vs. Light Therapy for Winter Depression
Trial registration {2a and 2b}.	Clinicaltrials.gov identifier: NCT03691792.
Protocol version {3}	Protocol Version 20, 01/31/2022
Funding {4}	This study is funded by the National Institute of Mental Health (R01MH112819-01A1).
Author details {5a}	Kelly J. Rohan and Pamela M. Vacek, University of Vermont; Peter L. Franzen, Kathryn A. Roecklein, Greg Siegle, and David Kolko, University of Pittsburgh; Teodor T. Postolache, University of Maryland
Name and contact information for the trial sponsor {5b}	National Institute of Mental Health, 6001 Executive Boulevard Room 6200, MSC 9663, Bethesda, MD 20892-9663; phone: 1-866-615-6464 (toll-free), Email: nimhinfo@nih.gov
Role of sponsor {5c}	The funding agency has/had no role in the design, collection, management, analysis, or interpretation of data; the writing of the manuscript; or the decision to submit the study protocol for publication. The funding agency has no ultimate authority over any of these activities.

## Introduction

### Background and rationale {6a}

Winter seasonal affective disorder (SAD) is a subtype of recurrent depression involving major depressive episodes during the fall and/or winter months that remit each spring [1]. SAD characterizes 10–20% of recurrent depression cases [2, 3]. SAD prevalence increases with latitude in the USA, ranging from 1.4% in Florida to 9.9% in Alaska [4, 5]. Averaging across latitude, SAD affects an estimated 5% of the US population—over 14.5 million Americans [6]. Seasonality is a dimensional construct that is normally distributed in the general population, with full-blown, clinical SAD symptoms representing the extreme variant along the human seasonality continuum [7, 8].

Given its recurrent course, the cumulative effect of SAD across the lifetime makes it an important public health challenge. Data on over 600 SAD patients at the National Institute of Mental Health (NIMH) Seasonal Studies Program 1981–2001 suggest that an untreated fall/winter major depressive episode persists for an average 4.9 ± 1.4 months before spontaneous springtime remission (N. E. Rosenthal, personal communication, May 24, 2005). A multisite study of 1042 SAD patients reported a mean age for onset of 27.2 years and, on average, 13.4 past fall/winter major depressive episodes [9]. These data suggest that SAD patients spend over 40% of the year struggling with substantial depressive symptoms that adversely affect the family and workplace during most years, beginning in young adulthood. In addition, SAD is associated with wintertime impairment in overall health, emotional well-being, daily activities, social activities, and pain [10].

This confirmatory efficacy R01 trial focuses on two non-pharmacological treatments that each work for some SAD patients: light therapy (LT; i.e., daily exposure to bright artificial light during the symptomatic months), and SAD-tailored group cognitive-behavioral therapy (CBT-SAD) [11]. Substantial evidence supports LT as an efficacious acute SAD treatment [12]. In our previously completed R01-level efficacy trial enrolling 177 adults with SAD, CBT-SAD and LT were very similar in terms of symptom improvement and the proportions in remission at post-treatment (47.6% CBT-SAD, 47.2% LT) [13]. However, two winters later, CBT-SAD was associated with significantly fewer depression recurrences (27.3% CBT-SAD, 45.6% LT) and lower depression severity than LT [14]. These findings suggest that slightly more than one-quarter of those treated with CBT-SAD and slightly less than half of those treated with LT have a depression recurrence, highlighting the need for reliable strategies for precision medicine in clinical practice. In the current project, we apply the experimental therapeutics approach to determine how each treatment works and to identify the best candidates for each. Consistent with the NIMH’s focus on biological endpoints, we will ascertain whether theoretically derived candidate biomarkers of each treatment’s target and effect are prescriptive of better outcomes in that treatment vs. the other.

#### Light therapy effectively treats acute sad and correction of circadian phase is mechanistic of its antidepressant effects

Light therapy (LT) is the established, most researched, acute SAD treatment. Meta-analyses show that 53% of SAD patients fully remit (and 47% do not) over an LT trial [15]. Clinical practice guidelines recommend solo LT as a first-line acute SAD treatment and reinitiating daily LT each fall from first symptom until spontaneous springtime remission [16, 17]. However, only 41% of those treated acutely with LT continue using it over subsequent winters [18]. Several studies support circadian rhythm disturbances in SAD patients, with their circadian phase occurring either later (phase delayed) or, less commonly, earlier (phase advanced) compared to their euthymic circadian phase position following remission with LT [19–22] or to healthy individuals in winter [19, 22]. As restoring the correct phase of circadian rhythms is supported as LT’s mechanism of action [15, 23–25], circadian phase measures are established biomarkers of LT’s target and effect. We will use the best established method, dim light melatonin onset (DLMO), along with wrist actigraphy, an objective measure of sleep/wake patterns, to calculate Phase Angle Difference (PAD) = DLMO–mid-sleep [21].

#### Normalizing retinal subsensitivity might also be mechanistic of LT

Reduced retinal sensitivity to light in winter is associated with SAD [26]. The melanopsin pathway, from the retina to the brain, is an initial input pathway that could lead to downstream changes in circadian entrainment [20], duration of nocturnal melatonin release [27], and seasonal variation in monoamine concentration [28]. In a diathesis-stress model of SAD, low winter light conditions may combine with a genetic diathesis (photopigment genetic variation) to cause melanopsin-driven retinal subsensitivity, leading to an insufficient central nervous system (CNS) signal to either properly entrain the circadian clock, or for non-circadian, direct effects of light on the CNS [29]. A reduced post-illumination pupil response (PIPR) [26, 30], i.e., a smaller net difference in pupil response to red vs. blue light reflex trials, is an established biomarker of melanopsin-driven retinal subsensitivity. Melanopsin-containing retinal cells are uniquely sensitive to blue light, leading to a sustained pupillary constriction when stimulated. The PIPR is a sustained pupillary constriction after lights off and has been linked to melanopsin cell firing through intracellular recording in primates, even when inputs from rods and cones are pharmacologically blocked [30]. The PIPR provides an elegant way to isolate melanopsin contributions from those of rods and cones, making the PIPR a viable candiate biomarker of LT’s target and effect. In a comparison of individuals with SAD and controls with no history of depression tested in winter and summer, the SAD group had a lower PIPR in winter than in summer, whereas controls did not differ across the seasons [31]. In addition, the SAD group had a lower PIPR than controls in winter, but the groups did not differ in summer.

#### CBT-SAD effectively treats sad, lowers risk of recurrence, and engages a cognitive target

CBT-SAD uniquely engages a potentially SAD-specific cognitive vulnerability as a target: seasonal beliefs, i.e., rigid and maladaptive beliefs about the seasons and light availability [32]. Seasonal beliefs distinguish between individuals with SAD vs. non-seasonal unipolar depression [32, 33]. Seasonal beliefs change significantly more over the course of CBT-SAD than during LT, and greater change towards more flexible seasonal beliefs during treatment mediates both improvements in depression symptoms during active treatment [34] and lower depression scores one and two winters following treatment [35]. This provides evidence that engagement of the seasonal beliefs target mediates CBT-SAD’s acute antidepressant effects and its enduring benefits over LT.

#### Targets and biomarkers of CBT-SAD’s response

Consistent with the experimental therapeutics perspective, the proposed work intends to map seasonal beliefs onto biomarkers of CBT-SAD’s target and effect. Evidence supports that CBT for non-seasonal depression works via altering mechanisms of maladaptive thought patterns. Sustained pupil dilation to emotional words, which indexes prefrontal control mechanisms associated with emotion regulation [36, 37], is a potential biological mechanism of CBT response. Sustained frontal gamma-band EEG responses to emotional stimuli indicates sustained elaborative emotional processing and potentially sustained regulatory effort [38], is a biomarker of depression [38], and represents another candidate biomarker of CBT for depression’s target and effect. In SAD, we propose that maladaptive thoughts are dominated by seasonal- and light-related themes, matching the content of seasonal beliefs that appear mechanistic of CBT-SAD. Therefore, we will explore whether pupil dilation and EEG responses to seasonal words change more during CBT-SAD than LT (target engagement) and whether these changes are prescriptive of better outcomes in CBT-SAD than in LT, in the way that seasonal beliefs are. Such findings would indicate that patients who do not recur following CBT-SAD are less emotionally reactive to seasonal stimuli, thereby engaging in less elaborative processing of these stimuli and requiring less prefrontal control to regulate their emotional responses to them.

#### Strategies for managing SAD recurrences following a first-line treatment are lacking

A long-term perspective on mood status in future winters reflects SAD’s recurrent nature, consistent with the central public health challenge to prevent recurrence. SAD’s predictable, seasonal course of onset and remission “uncouples” the treatment and prevention phases of randomized clinical trials spanning multiple years. Acute treatment response and recurrence are two different outcomes, both worthy of attention. Although the field lacks an accepted SAD “treatment failure” definition, we argue that recurrence is a more clinically meaningful benchmark than post-treatment non-remission status. It is inarguably a poor outcome if a SAD patient achieves post-treatment remission but develops a recurrence the next winter. Conversely, it is a favorable outcome if a SAD patient does not fully remit at treatment endpoint, but reinitiates LT or uses cognitive-behavioral strategies early the next year to prevent recurrence. No algorithms exist to guide clinicians when a patient recurs following a first-line SAD treatment. Even basic non-responder trials are scarce, with only a few small-scale SAD studies (Ns = 13–16) switching or augmenting with tryptophan following nonresponse to LT [39, 40].

### Objectives {7}

Our goal is to inform evidence-based guidelines for personalized medicine using CBT-SAD and LT, specifically for determining [1] which patients are best suited for which type(s) of treatment in terms of recurrence prevention, and [2] whether the selected treatment engages its intended target. We will ascertain whether theoretically derived candidate biomarkers of each treatment’s target and effect are prescriptive of better outcomes in that treatment vs. the other. We will examine the established chronobiological biomarker of LT’s target and effect, PAD, and explore the PIPR as a candidate biomarker of melanopsin-driven retinal subsensitivity with specificity to LT. We will use self-reported seasonal beliefs as the established target and mechanism of CBT-SAD and also map this construct onto theoretically relevant biomarkers of reduced emotional reactivity to seasonal stimuli, pupil dilation and sustained frontal gamma band EEG responses to seasonal words. In addition to determining whether these interventions engage associated change mechanisms, we will test the efficacy of a “switch” decision rule upon recurrence the next winter to inform clinical decision-making for managing SAD recurrences following a first-line treatment.

In this confirmatory efficacy R01, adults with SAD (target *N* = 160) will be randomized to two equal-probability 6-week treatment conditions in winter 1: CBT-SAD or LT. We will follow subjects in winter 2 and if a depression recurrence occurs, we will cross them over into the alternate treatment (i.e., switch to CBT-SAD upon recurrence after LT, switch to LT upon recurrence after CBT-SAD). All subjects will be assessed at follow-up in winter 3. Biomarker assessments will occur at pre-, mid-, and post-treatment in winter 1; at winter 2 follow-up (and again at mid- and post-treatment for those with depression recurrences crossed-over into the other treatment); and at winter 3 follow-up. This work will yield longitudinal data on targets, their engagement, and stability, thereby increasing the impact of future dissemination efforts.

Specific aims are:

1. To confirm the superiority of CBT-SAD over LT on depression recurrence status (the primary outcome)
2. To elucidate differential mechanisms of acute treatment effects in CBT-SAD vs. LT by examining candidate biomarkers as potential mediators and to assess whether biomarkers at baseline, changes during treatment, and stability of changes over time are predictive of acute and/or long-term outcomes (recurrences)
3. To compare a switch to CBT-SAD for those who recurred after LT to a switch to LT for those who recurred after CBT-SAD. We expect that a switch from LT➔CBT-SAD will show advantage over a switch from CBT-SAD➔LT in depression recurrences at winter 3, based on prior findings for greater durability in CBT-SAD
4. To examine mediators of the effect of CBT-SAD on outcomes at winter 2 and winter 3

### Trial design {8}

**Fig. 1:**
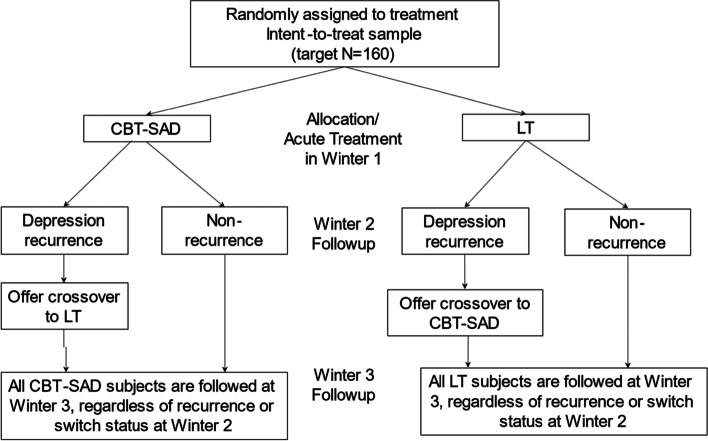
Anticipated participant flow in the randomized clinical trial

This is a head-to-head confirmatory efficacy trial comparing CBT-SAD and LT (with equal-probability allocation) designed to test superiority of one treatment over the other in depression recurrences at winter 2 follow-up. Participants with recurrences at winter 2 follow-up will be offered the opportunity to cross-over into the alternate treatment. All participants are followed-up at winter 3 (Fig. 1).

## Methods: participants, interventions, and outcomes

### Study setting {9}

This is a single-site trial taking place in the principal investigator’s (K.J.R.) mood disorders and seasonality research laboratory at the University of Vermont, Department of Psychological Sciences, Burlington, VT, USA.

### Eligibility criteria {10}

We will use the same inclusion/exclusion criteria from our previous R01 clinical trial, as they balance rigor and relevance for community samples and yielded a sample of the proposed size. Inclusion criteria are as follows: (a) principle DSM-5 diagnosis of Major Depression, Recurrent, with Seasonal Pattern on the Structured Clinical Interview for DSM-5 Axis I Disorders— Research Version (SCID-5-RV) [41], (b) meet Structured Interview Guide for the Hamilton Rating Scale for Depression—Seasonal Affective Disorder Version (SIGH-SAD) [42] criteria for a current SAD episode (total score ≥ 20 and ≥ 5 on the 8-item atypical subscale), and (c) no use or stable use of antidepressants (i.e., a consistent dose of the same medication maintained for ≥ 4 weeks with no plans to change), and (d) medical clearance to participate on the basis of physical exam/medical work-up at the University of Vermont Clinical Research Center (CRC). [Note regarding (c): In the last R01 study, 25.4% of subjects were taking antidepressants at baseline, and only 1 subject reported a medication change during treatment]. Exclusion criteria are as follows: (a) current or past LT or CBT for SAD, (b) current or past bipolar disorder or psychotic disorder, (c) current substance use disorder beyond mild severity, (d) acute and serious suicidal intent, (e) planned absences of > 1 week from the area during the projected treatment interval, (f) history of conditions that are known contra-indications to LT, including conditions associated with toxicity of bright light to the retina (i.e., macular degeneration or any retinopathy), (g) photosensitizing reaction to any current medication (e.g., rash upon exposure to sunlight); and (h) positive laboratory findings for hypothyroidism at medical screening/work-up visit.

#### Screening and enrollment procedures

Participants will be recruited from media advertisements, referrals from local physicians and mental health clinics, and community outreach. Interested volunteers will undergo a brief phone screening to assess basic inclusion/exclusion criteria, including assessment of major depressive episode criteria for the previous winter. Those who appear eligible will be invited to review the informed consent form, and if they consent to the study, to proceed with diagnostic interview, the Structured Clinical Interview for DSM-5 Axis I Disorders—Research Version (SCID-5-RV) [41], administered by the P.I. or a trained clinical psychology graduate student research assistant. If the SCID diagnosis is Major Depressive Disorder, Recurrent, with Seasonal Pattern (inclusion criterion a) and there are no comorbid disorder exclusions (exclusion criteria b and c); the participant will be asked to attend the medical screening/work-up where they will undergo a physical examination and medical chart/history review with a physician assistant, vital signs are collected (blood pressure, pulse, height, and weight), and blood is drawn for thyroid panel (TSH, and free T4). If no medical exclusion is identified (inclusion criterion d and no documented evidence of exclusion criteria b, c, or f on the basis of medical record review), the participant enters the symptom monitoring phase of the study. The SIGH-SAD will be administered by a trained research assistant every other week until SIGH-SAD criteria for a current SAD episode (inclusion criterion b) are met. When a potential participant meets SIGH-SAD criteria for a current SAD episode, they will be asked to attend the pre-treatment assessment, at the conclusion of which, the participant is actively enrolled and randomized. After the first week of February, SIGH-SADs will be discontinued for any potential participant not meeting threshold for a SAD episode because: (1) in our experience, after this date, there is low likelihood of developing a full threshold episode in that winter, and (2) insufficient time remains to ensure completion of the pre-treatment assessment procedures and the 6-week treatment phase before the possibility of spontaneous springtime remission.

### Who will take informed consent? {26a}

The phone screen step includes a Consent Process Documentation for Phone Screen completed by the research assistant performing the phone screen, which documents the potential subject verbally consented to participate in a phone screen for the project. Those eligible on phone screen are invited to the diagnostic interview (SCID-5-RV). In the interim, they are sent an appointment reminder and an electronic version (PDF) of the informed consent document to review. The P.I. and graduate student research assistants who conduct the diagnostic interviews are the ones who seek informed consent. When the potential subject presents, we first go over the informed consent document in detail and answer any questions. We explain that participation is voluntary and they may decline to participate, take more time to think it over, or consent and proceed with the diagnostic interview. If they decide to consent, the subject and P.I. or designee both sign and date the consent document and proceed with the diagnostic interview. We then complete a Consent Process Documentation form for this protocol. The consent form was completed in paper form from during the fall/winters of 2018-2019 and 2019-2020. Effective for the fall/winter of 2020-2021 and beyond, due to the COVID-19 pandemic, we used HIPPA-compliant videoconference to review the consent form with potential volunteers and obtained written consent via REDCap electronic data capture tools hosted at the University of Vermont.

### Additional consent provisions for collection and use of participant data and biological specimens {26b}

This trial does not involve collecting biological specimens for storage.

## Interventions

### Explanation for the choice of comparators {6b}

The CBT-SAD protocol used in this study is justified because it is the only psychotherapy treatment for SAD with data to support its efficacy. The comparator LT condition is justified because LT is the established acute SAD treatment. Antidepressant medications represent another first-line SAD treatment; however, this trial was funded under an FOA focused specifically on confirming the efficacy of non-pharmacologic treatments. Although some people might rather be in a trial where there is the possibility of receiving the combination of CBT-SAD and LT, administered concurrently, we did not include a combination arm in the design. This is based on our prior study that tested the combination of CBT-SAD+LT against solo LT, solo CBT-SAD, and a wait-list control group. All three active treatments improved winter depression symptoms as compared to the wait-list control over 6-weeks, with no significant differences between them [43]. However, at follow-up the next winter, solo CBT-SAD was superior to solo LT on recurrences and depression severity, and these differences survived adjustment for re-treatment in the interim [44]. In contrast, the combination group was superior to solo LT only on recurrences, and this difference became non-significant after adjusting for re-treatment [44].

### Intervention description {11a}

#### Timing of the treatments

Given SAD’s inherent seasonality, recruitment will begin September 1 and participants will be enrolled on a continuous basis as they qualify. The second week of February will be the latest treatment start date. Similar timing procedures worked successfully in our prior studies. Our data suggest that the treatment groups grew at comparable rates over time with no significant differences in the timing of treatment initiation or cessation between the groups, and that treatments were completed during the winter.

#### Cognitive-behavioral therapy (CBT-SAD)

The P.I. (K.J.R.) tailored traditional CBT for depression to address the special needs of the SAD population in developing the “Coping with the Seasons” protocol and its manual [11]. CBT is placed in a seasonality framework with the rationale addressing the role of environmental changes as well as cognitions and behavior in symptom onset/maintenance. Traditional CBT elements of behavioral activation and cognitive restructuring are framed as ways to improve coping with winter. Some cognitive restructuring specifically focuses on challenging negative thoughts related to the winter season, light availability, and weather. A recurrence-prevention component addresses early identification of negative anticipatory thoughts about winter and SAD-related behavior changes, using the CBT skills learned to cope with future winter seasons, and development of a personalized relapse-prevention plan. As in prior studies, the CBT-SAD protocol includes 90-min. CBT sessions twice a week over 6 weeks (total of 12 sessions and 18 h) in small groups (typically 4–8 patients per group). CBT-SAD sessions were conducted in-person until March 2020, when we moved them to HIPPA-compliant video conference platform due to the COVID-19 pandemic. In our prior R01, CBT-SAD attendance was quite good, mean = 9.1 sessions (SD = 3.5). Seventy-three of 88 (83%) attended 7 or more sessions, and 56/88 (64%) attended 10–12 sessions. To accommodate unpredictable schedules, if a patient misses a session, there is opportunity to “catch-up” with group facilitator before the next session and to make up the session in another concurrent CBT-SAD group; both are documented.

#### Therapists

Six trained clinical psychology graduate student research assistants will facilitate the CBT-SAD groups, supervised by the P.I. (K.J.R.). Each group will be led by a pair of group facilitators. The P.I. will provide therapists with session-by-session training in the manual content prior to their providing any treatment. Subsequently, PI Rohan will review audiotapes of each CBT-SAD session and will discuss these sessions in weekly 1-h group supervision meetings with each pair of facilitators focused on [1] discussion of general issues in therapy, participant progress, and the treatment protocol, and [2] the P.I. providing detailed feedback on the prior two sessions. Supervision and training meetings occurred in person until March 2020 when we shifted to videoconference due to the pandemic, with all parties participating from a private office to protect confidentiality. These training procedures were successfully used to train two community therapists in our prior R01 study, with no significant differences between P.I. Rohan and the other two therapists in post-treatment outcomes (all ps > .50). The multi-component supervision protocol reflects what works best in the field [45].

#### Light therapy (LT)

Participants attend an initial instructional session that follows a script to explain the treatment rationale and assembly/position of the light box. These will be conducted by the (unblinded) P.I. in small groups of 2–4 subjects for efficiency. Effective fall/winter of 2020–2021, the P.I. posted a video recording of the instructional session on the lab’s private YouTube channel for those randomized to light therapy to watch remotely, due to the COVID-19 pandemic. In their homes, LT participants will use the SunRay (SunBox Company, Gaithersburg, MD), a standard device (23” × 15½”× 3¼”) with an ultraviolet shield that emits 10,000-lux of cool-white fluorescent light to the retina when the user is within 18 inches. Each of the 6 weeks, participants will complete the Light Therapy Side Effects Questionnaire used in our prior studies to assess the presence and severity of any side effects attributed to LT and self-reported times for onset of sleepiness, onset/offset of actual sleep, and desired times for onset/offset of sleep over the previous week. Side effects associated with LT are generally mild and can include headache (19%), eyestrain (17%), and feeling “wired” (14%) [46]. Although individuals typically respond within 4 weeks of initiating LT or not at all [16], our LT protocol will be maintained for 6 weeks to match CBT-SAD’s duration. To circumvent ethical concerns of discontinuing LT during the winter (when relapse is likely) [1], participants are encouraged to keep using the light box and bring it to the lab for servicing in May (to check the bulbs against a photometer and download adherence data). In an effort to reduce access to a suitable device as a potential confound, we will offer access to our light boxes to LT participants in winters 2 and 3 and to CBT-SAD participants crossed into LT in winter 2 for use in winter 3.

During each week of LT administration, the P.I. will contact Co-I (T.T.P.) via phone to review each LT subject’s weekly SIGH-SAD scores to date and responses on the Light Therapy Side Effects Questionnaire. We successfully used this type of telephone supervision for the LT condition in our prior studies. Because direct comparisons show morning LT is a more potent antidepressant than evening LT [15], and as articulated above, it is assumed that that morning light manifests its effects via correcting a pathological circadian phase-delay in SAD, we will initiate LT at 30-minutes in the morning, first thing upon awakening, to be completed between the hours of 0600 and 0900 (i.e., in the phase advancing portion of the phase response curve to light) [47]. After the first week, Dr. Postolache will recommend individually tailored adjustments to the duration of light use per our treatment algorithm to maximize response and reduce any reported side effects. Incremental changes in the duration of LT are initiated upon an insufficient response to light, defined as a < 30% reduction in SIGH-SAD at the end of week 1, < 50% reduction in SIGH-SAD at the end of week 2, or not fulfilling SIGH-SAD remission criteria at the end of week 3 and beyond. In these cases, if the side effect profile permits, the duration of LT is increased in steps of 15 min/day up to a maximum of 2 h/day. Decrements in LT are based on side effects and consist of decreasing the duration of LT in steps of 15-min/day. LT is temporarily halted for severe side effects (e.g., severe migraines) and restarted the following day with a reduced duration (50%), which is slowly increased to tolerance.

There are two concerns related to circadian phase-shifting induced by light: side effects of the phase-shift and reduced antidepressant effectiveness if addressing the phase-shift. In a re-analysis of Eastman et al.’s data [23], evening light treatment for SAD phase-delayed and morning light treatment phase-advanced the temperature minimum (Tmin) by 1 h [48]. Early awakenings and/or late afternoon or early evening sleepiness sometimes reported by patients could be the result of a circadian phase-advance induced by the morning administration of LT. If this occurs, the duration of the morning LT is reduced (if side effects present) and/or adjunct evening light is added. The P.I. will contact LT participants to convey any recommended changes to their LT prescription. These procedures for weekly adjustments to the daily duration of LT replicate those used in our previously completed R01.

### Criteria for discontinuing or modifying allocated interventions {11b}

We have operationalized decision rules to identify any participant who is escalating in severity during the acute treatment phase that will be uniformly applied across treatment conditions. These subjects will be removed from the treatment protocol and referred for SAD treatment outside the study. First, any participant who demonstrates one of the serious adverse events described below (i.e., serious side effects to light therapy, mania or hypomania, or significant suicidal intent) will be removed from the treatment protocol. Second, participants who fall outside of the acceptable range for increasing SIGH-SAD scores (i.e., an increase of 12-points or more over pre-treatment SIGH-SAD score) for 2 consecutive or nonconsecutive weeks over the treatment phase will be removed from the treatment protocol. To operationalize this definition, we used prior data to generate the means and variability for the weekly SIGH-SAD scores for concurrent wait-list controls during the 6-week waiting phase when they were untreated [43]. The means and SDs were relatively consistent over the 6 weeks; therefore, we calculated the mean SIGH-SAD and its SD over the 6-weeks for the WLC condition (24.48 ± 5.78). Therefore, a 12-point increase over pre-treatment SIGH-SAD indicates a SIGH-SAD score that is 2 SDs above the typical variance observed for our wait-list controls. The reason that we specify 2 weeks is to minimize the chance that a temporary stressor contributed to a temporary exacerbation as opposed to a true treatment nonresponse. Even if removed from protocol, we will include all cases in the analysis and we will attempt to collect data from all participants, at each time point as the primary analysis is an intent-to-treat analysis of all randomized cases.

Related to these two instances where we will remove a participant from the treatment protocol are cases where participants initiate outside treatment on their own during their study treatment. To assess the initiation of treatments outside of the study, we have added relevant questions to the beginning of each SIGH-SAD assessment. We will uniformly ask the question, “Have you started any new treatments outside of the treatment you are receiving in this study such as light therapy, talk therapy, or medications?” If a participant responds affirmatively, they will be queried by the interviewer for the details of this non-study treatment, which will be recorded. If a new treatment has been initiated, these cases will uniformly be considered as withdrawn from treatment protocol.

Study records include SIGH-SAD scores at each timepoint for each participant in the study. The project coordinator will monitor these records weekly to determine if any participant has met the criteria for protocol withdrawal. The PI will refer these cases for more intensive individual treatment from a local provider with training in treating SAD/depression. The project coordinator will also monitor weekly responses to the queries about initiating a new, non-study treatment to detect any treatment failures. Participants are informed that their progress over the study will be closely monitored; and, if at any time symptom severity escalates, the P.I. will discuss with them the possibility of referral for outside treatment.

To summarize, participants who experience a severe adverse event or meet our criteria for an excessive exacerbation in SIGH-SAD score during treatment will be removed from the treatment protocol and referred for outside treatment. Participants who decide to seek any outside treatment on their own will be coded as voluntarily withdrawn from protocol. According to intent-to-treat (ITT) principles, such participants will be encouraged to attend ongoing study monitoring (SIGH-SADs) to gauge their symptom severity and to participate in the follow-ups per the study assessment schedule. They will be included in ITT analysis as randomized, and also analyzed in an actual-treatment analysis. If initiation of outside treatment occurs, this event will be coded for in the database. Decisions about whether to consider this as a moderator of outcome, will be made in consultation with the Co-I study biostatistician (P.M.V.) based on how frequently this event occurs.

### Strategies to improve adherence to interventions {11c}

#### Patient adherence to treatment

Patient adherence to CBT-SAD will be assessed via session attendance. Each therapist will keep a session attendance record, submitted to the Project Coordinator at the end of each treatment week. If a participant misses a CBT-SAD session, the group facilitators attempt to contact them prior to the next session to discuss their absence and encourage continued attendance. In addition, each therapist will keep session-by-session records of homework completion (0 = no homework, 1 = partial completion of homework, 2 = completion of homework). Beginning in session 2, each CBT-SAD session includes a thorough review of the homework assigned at the end of the prior session. To assess homework completion from the patient perspective, weekly during the 6 treatment weeks, CBT-SAD subjects will rate their frequency of completing the homework assignments, on average, over the past week on a 5-point scale (0 = never, 1 = less than 1 day per week, on the average, 2 = 1 day per week, on the average, 3 = 2 or 3 days per week, on the average, 4 = more than 3 days per week) [49].

Adherence to the LT prescription will be objectively monitored with a data logger to monitor time turned on and the duration before being switched off. Data will contain time and date stamps. When returned to the lab, data will be downloaded by USB connection to PCs for analysis. Data will be sampled every second to minimize the need for data reduction. During active LT, each participant is asked to keep a diary of light therapy use to document state and end times by date so the P.I. can track self-reported compliance each week. If a participant reports light therapy durations that are shorter than what was recommended, the P.I. will contact them to discuss it and encourage using LT at the currently prescribed duration to maximize efficacy. We also hold-off on making a weekly LT duration increase if the participant has been compliant with the current dose and instead attempt to address compliance.

#### Treatment integrity

As in our prior trials, we will use a modification of the NIMH Collaborative Study Psychotherapy Rating Scale (CSPRS) [50, 51], which measures the extent of specific therapist behaviors. Original CSPRS items assessing CBT intervention components were retained with items added to assess components specific to our CBT-SAD manual. The CSPRS language was modified to reflect group versus individual therapy. Items pertaining to clinical management were rephrased to reflect LT (as opposed to imipramine). A random sample (25%) of session audio-recordings will be selected from each condition (CBT-SAD or LT) and session number and study therapist (in the case of CBT-SAD). Two trained clinical graduate students, blind to condition and session number, will rate the selected audio-recorded sessions. Inter-rater reliability and success at discriminating between the content of the CBT-SAD and LT conditions and between individual session numbers within the CBT-SAD condition will be computed.

### Relevant concomitant care permitted or prohibited during the trial {11d}

As described in the inclusion/exclusion criteria, stable use of antidepressant medications is permitted during the trial.

### Provisions for post-trial care {30}

One human subjects issue in this work is balancing the need for treatment integrity over the follow-up interval with the need for re-treatment. A rigorous test of our aims requires steps to increase the likelihood that treatment over the follow-up interval is consistent with study treatment (i.e., that LT subjects are encouraged to continue light therapy each fall/winter and that CBT-SAD subjects are encouraged to keep using the skills learned in CBT-SAD on their own without a therapist). However, we recognize that we cannot ethically proscribe treatment in the interim and that we are accountable for directing them to resources if there is a need for re-treatment. We have developed an approach that attempts to balance these issues. (By design, one way we will deal with this is the cross-over at winter 2 for subjects who evidence a SAD recurrence at the winter 2 follow-up).

As a step to increase the likelihood that treatment in the interim is consistent with initial study treatment, we will use standardized procedures for instructing participants at the end of study treatment. The first week of September, we will mail standardized letters, signed by the PI, to all participants treated the winter before prompting them to resume study treatment and to pursue new treatments only if needed. Both the CBT-SAD and LT letters include the same, standardized safety net in providing contact information for local mental health centers and treatment providers should they require more formal treatment. The letter to CBT-SAD-treated participants encourages them to keep using the skills learned in the CBT-SAD treatment on their own (without a therapist). The letter to LT-treated participants encourages them to re-initiate daily LT upon onset of the first depressive symptom in the coming fall/winter by either [1] borrowing one of our light boxes or [2] purchasing a device. Should they wish to purchase their own light box, we provide contact information for LT manufacturers with a list of specifications to match our devices (i.e., full-sized units emitting 10,000-lux cool-white light through UV filter). For either scenario, we ask the participant to identify a professional to whom they could turn in the event of side effects (since we will no longer monitor side effects and make prescription adjustments, as we did during acute treatment). LT participants may choose whether or not to have the PI provide a signed, generic letter that the participant may submit to his or her health insurance company to seek reimbursement for the cost of a light box. Participants crossed from CBT-SAD to LT at winter 2 will receive the standard LT letter the next September (winter 3), and participants crossed from LT to CBT-SAD will receive the standard CBT-SAD letter the next September.

### Outcomes {12} (Table 1)

#### Primary outcome: structured interview guide for the Hamilton Rating Scale for Depression—Seasonal Affective Disorder Version (SIGH-SAD

The SIGH-SAD (42) includes the 21-item Structured Interview Guide for the Hamilton Rating Scale for Depression (HAM-D) and a supplementary 8-item subscale to assess atypical depressive symptoms associated with SAD. A trained rater, blind to treatment condition, will administer the SIGH-SAD at pre-treatment, treatment weeks 1–5, post-treatment, and winter follow-ups. The rater will always begin by asking if any new treatment other than the study treatment has been initiated and if any medications have changed. This will be coded in the database and considered as a possible covariate. A second blinded rater will rate audio-recordings of the SIGH-SADs to assess inter-rater reliability. The depression recurrence (primary outcome) criteria define SAD episode onset or recurrence [62] and were used in our prior trials: total SIGH-SAD score ≥ 20 + HAM-D score ≥ 10 + atypical score ≥ 5. Remission at treatment endpoint is classified by satisfying one or both of the following criteria [62]: (1) pre- to post-treatment reduction in total SIGH-SAD score ≥ 50% + HAM-D score ≤ 7 + atypical score ≤ 7, OR (2) HAM-D score ≤ 2 + atypical score ≤ 10. The same criteria will be applied to winter follow-up remission status by comparing follow-up to pre-treatment scores. We will follow our published SIGH-SAD protocol for item scoring rules, rater training procedures, and resolving rater discrepancies [63]. SIGH-SADs were administered live until March 2020 when we shifted to HIPPA-compliant videoconference due to the COVID-19 pandemic.

**Table 1:** Measures and assessment schedule

Measure	Construct assessed	Metric	Pre-TX	Weekly during TX	Mid-TX*	Post-TX*	Winter 2	Winter 3
SIGH-SAD [42] (see description)	SAD recurrence (primary outcome) and remission status, symptom severity	Categorical recurrence status, categorical remission status, score	X	X	X	X	X	X
Beck Depression Inventory-II (BDI-II) [52]	Depression symptom severity, remission status (BDI-II ≤ 8)	Score, categorical remission status	X	X	X	X	X	X
Beck Anxiety Inventory [53]	Anxiety symptom severity	Score	X		X	X	X	X
Dim light melatonin onset (DLMO)	Circadian timing	Clock time (military)	X			X	X	X
Wrist actigraphy and Pittsburgh Sleep Diary [54]	Sleep & circadian timing; daytime activity	Mean mid-point of sleep; mean daily activity count	X			X	X	X
^†^Phase angle difference (PAD)	Circadian timing	DLMO–mid-sleep (from actigraphy)	X			X	X	X
^†^PIPR	Post-illumination pupil response	Net PIPR (red–blue light response)	X		X	X	X	X
^‖^Pupil dilation and sustained frontal gamma-band EEG responses to negative and winter words	Prefrontal engagement as it relates to emotion elaboration and regulation	Mean pupillary and sustained frontal gamma responses 2–10 s following emotional word onset	X		X	X	X	X
^‖^Seasonal Beliefs Questionnaire (SBQ) [32]	Maladaptive thoughts about the seasons and light availability	Score	X		X	X	X	X
^‖^Dysfunctional Attitudes Scale-Form A (DAS) [55]	Depressive schemas	Score	X		X	X	X	X
^‖^Behavioral Activation for Depression Scale (BADS) [56]	Self-reported behavioral activation	Score	X		X	X	X	X
^†^Composite Scale of Morningness (CSM) [57]	Chronotype	Score	X		X	X	X	X
Treatment Credibility Scale [58]	Credibility for the CBT-SAD and LT treatment conditions	Score	X		X	X		
Millon Clinical Multiaxial Inventory—Fourth Edition (MCMI-IV) [59]	DSM-5 personality disorders	Scale scores, possible DSM-5 personality disorders	X					
Quality of Life Enjoyment and Satisfaction Questionnaire (Q-LES-Q) [60]	Quality of life related to: physical health, mood, leisure time activities, social relationships, general activities, work (if applicable), household duties (if applicable), and school/coursework	Score	X			X	X	X
Sheehan Disability Scale (SDS) [61]	Mental-health related impairment in work, social life/ leisure activities, and home life/family responsibilities	Score	X			X	X	X
MOS SF-20 (62)	Health-related quality of life	Score	X			X	X	X
SCID-5-RV major depressive episode criteria	Recurrences between post-treatment and winter 2 and between winters 2 and 3	Categorical recurrence status					X	X

*Repeated in winter 2 if switched to the alternate treatment

^†^Candidate mediator of LT’s effects

^‖^Candidate mediator of CBT-SAD’s effects. TX-treatment

#### Secondary outcomes

The Beck Depression Inventory-Second Edition (BDI-II) [52] is included as another depression outcome measure. The Beck Anxiety Inventory (BAI) [53] was added to the protocol through an administrative supplement to the parent project to determine whether treatment effects generalize to anxiety. The following assess functional outcomes: Quality of Life Enjoyment and Satisfaction Questionnaire (Q-LES-Q) [60], Sheehan Disability Scale (SDS) [61], and the MOS SF-20 [64].

### Participant timeline {13}

**Fig. 2:**
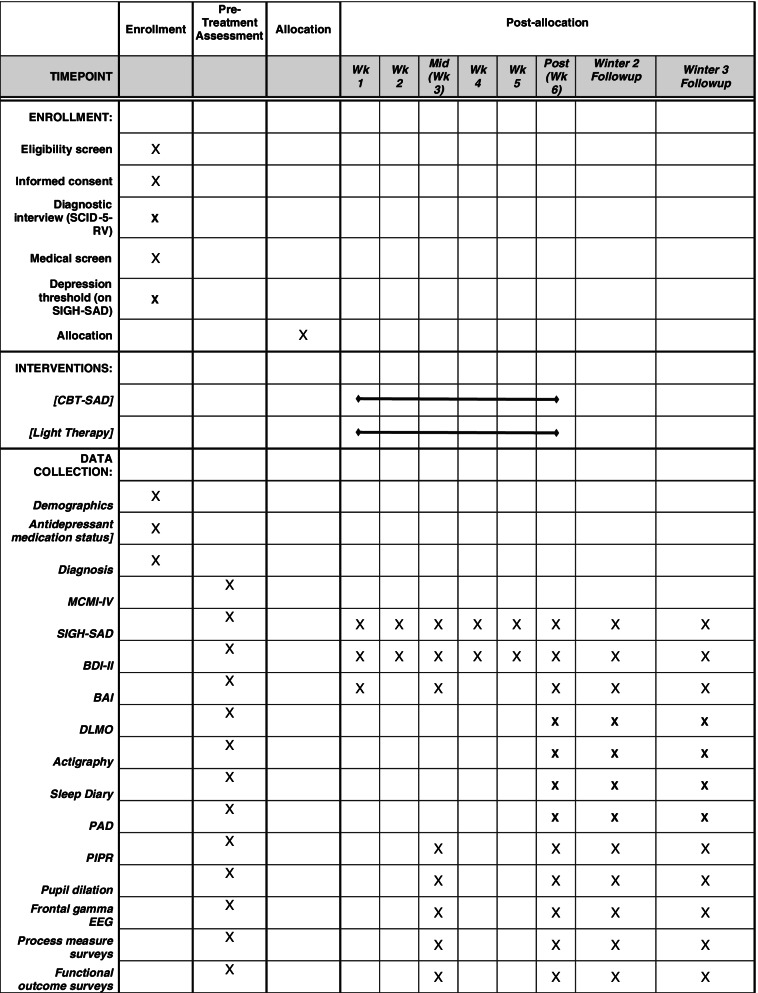
Schedule of enrollment, interventions, and assessments

The participant timeline is shown in Fig. 2.

### Sample size {14}

With a target *N* = 160 (80 subjects in each of the two treatments), the study will have 80% power to detect main effect differences of 22% (e.g., 28% vs. 50%) in winter 2 recurrence, which we expect at a naturalistic follow-up [14, 44]. Sample sizes for comparisons of winter 3 recurrence will depend on the number of recurrences at winter 2. Based on the recurrence rates observed in our prior study, we project that there will be about 37 recurrences in the LT group and 22 recurrences in the CBT-SAD group. This will provide 80% power to detect a difference in winter 3 recurrence of 35% (e.g., 60% vs. 25%) between CBT-SAD➔LT and LT➔CBT-SAD and a difference of 27% (e.g., 23% vs. 50%) between CBT-SAD and LT subjects without winter 2 recurrence. For biomarker comparisons, there will be 80% power to detect differences of 0.45 standard deviation (SD) in post-treatment values or in pre-post treatment changes between CBT-SAD and LT. Power for detecting relationships between biomarkers and outcomes were based on the odds ratio associated with an increase of 1 SD in the biomarker value or its change. For post-treatment remission, there will be 80% power to detect an OR of 2.2 in the CBT-SAD or LT group and a 2.4-fold difference in the ORs for the two treatments. For winter 2 recurrence, there will be 80% power to detect an OR of 2.2 in the LT group, an OR of 2.8 in the CBT-SAD group, and a 2.9-fold difference in their ORs. There was minimal attrition (4.5%) in our previous study, and we anticipate similar retention in this study. However, with attrition as high as 20%, there will be 80% power to detect a difference in winter 2 recurrence of 24% between CBT-SAD and LT and a difference of 39% in winter 3 recurrence between the two switch groups. Empirical sample size estimates^21^ indicate that the study will have at least 80% power to detect a mediated effect if the regression coefficient for the direct effect of treatment after adjustment for the mediator is at least .39 and the coefficients for the effect of treatment on the mediator and the effect of the mediator on outcome after adjustment for treatment are at least .26.

### Recruitment {15}

We intend to draw our sample from the combination of a multi-media advertising campaign and referrals from local health and mental health clinics beginning in September and continuing through early January of each year of recruitment. These recruitment strategies mirror those previously implemented by the P.I. to recruit cohorts of 40–50 new cases/year in prior studies. Our last study included 177 subjects recruited over 6 years; however, the first 2 years enrolled 24 subjects under an intramural pilot grant. The remaining 153 subjects were recruited under the R01 in 4 cohorts: 24, 33, 50, and 46. This averages 38 subjects/year, but we intentionally kept recruitment low the first two R01 years while we hired staff. While we had hoped to recruit cohorts of 50–55/year and complete recruitment in project years 1–3 for the current project, it proved difficult to recruit cohorts of that size, especially during the COVID-19 pandemic, and we extended recruitment into project year 4. Completion of all follow-ups through winter 3 will be possible with a one-year no-cost extension.

Our advertising campaign targets the major area newspaper, free local/community papers, neighborhood email list serves, Facebook, and radio advertisements across stations that cater to different demographics. To generate referrals, the P.I.’s laboratory maintains an updated database of all > 500 physicians (including all specialties), > 60 licensed Doctoral and Master’s-level psychologists, and > 20 private practice groups and community mental health centers in Chittenden County, the county where Burlington is located. Each September, we will perform outreach through these various sources and invite referral of SAD patients. We have found that these sites recognize our specific expertise in SAD (i.e., expertise they do not tend to have) and are glad to refer these cases for SAD-specific services through our research. Burlington, VT is home to the University of Vermont Larner College of Medicine, the largest employer of physicians in the State. Most Vermont residents centralize their healthcare needs in Burlington and are used to coming to Burlington for their healthcare. Therefore, we routinely do outreach to colleagues in the Medical School to generate referrals each year.

The rate of participant accrual is tallied, including a breakdown of rates for women and ethnic minorities, at the conclusion of each recruitment period (i.e., in the winter), and will be formally reviewed each study year by the P.I. and the DSM Board, and (at annual IRB review of the project) by the University of Vermont IRB. The number of participants who consented but did not qualify (i.e., were screened out at the medical screen or did not develop a SAD episode) and the number of qualified, enrolled participants who dropped out will also be analyzed to detect patterns of insufficient recruitment rates. These will be addressed aggressively with modified recruitment strategies so that we can meet our recruitment goals.

## Assignment of interventions: allocation

### Sequence generation {16a}

Participants will be randomly allocated to one of the two equal-probability conditions on a continuous basis as they qualify. Project biostatistician/co-investigator (P.M.V.) will design the schedule based on permuted random-size blocks of 4 and 8, stratified by three variables: [1] sex at birth, [2] comorbid diagnosis with two levels (present/absent), and [3] antidepressant medication status with two levels (on medication or not).

### Concealment mechanism {16b}

Only the project statistician will have access to the schedule and will prepare numbered, sealed envelopes with treatment assignments for each stratum.

### Implementation {16c}

Only the project coordinator, who will not conduct outcome assessments, will sequentially open the envelopes as subjects enroll to determine treatment group. Both the project statistician and an independent statistician will routinely check implementation of the randomization for accuracy.

## Assignment of interventions: blinding

### Who will be blinded {17a}

All study personnel other than the PI, the project coordinator, the project biostatistician, and CBT-SAD group facilitators will be masked to study treatment. Importantly, the trained research assistants who administer the primary outcome measure, the SIGH-SAD, will be blinded. Before each SIGH-SAD administration, the rater will inform the participant to answer the questions about symptoms over the past week without disclosing their treatment assignment. If a participant reveals their treatment assignment during a SIGH-SAD, the interview continues, but the rater marks the interview as unblinded and notes the time during the audio-recording when unblinding occurred. The project coordinator then erases the part of the interview when unblinding occurs, assigns a new (blinded) rater to rate the interview in place of the original interviewer, and avoids scheduling the participant with an unblended rater at future time points. Procedures for maintaining the blind in data management are discussed under the Data Management Plan, below.

### Procedure for unblinding if needed {17b}

The authors do not anticipate a requirement for unblinding because participants are aware of their treatment assignment. In the event of adverse events, the Principal Investigator is also aware of participant treatment assignments.

## Data collection and management

### Plans for assessment and collection of outcomes {18a}

#### Biomarker assessments

Pupillary and EEG data will be collected at pre-, mid-, and post-treatment in winter 1; at winter 2 follow-up (and for those who recur and are switched to the alternate treatment modality, again at mid- and post-treatment in winter 2); and at winter 3 follow-up (for all subjects, regardless of recurrence or switch status). The schedule for PAD is the same, with the exception of mid-treatment, due to subject burden and DLMO expense.

##### PIPR

The PIPR will be assessed using previously validated methods in SAD [26, 31]. The PIPR metric is calculated as the difference between pupil responses to red and blue light, as only responses to blue light reflect the circadian system. Due to daily variation in retinal responsivity, participants will undergo PIPR testing only during the daily plateau in responsivity. Stimuli are electronically controlled via light emitting diodes within the DP2000 Neuroptics (Irvine, CA) pupilometer goggles. Stimuli are 22.68 nm full width half-maximum (FWHM; 467.7 nm blue light) and 15.78 nm FWHM (632.9 nm red light) and both are calculated to yield a retinal irradiance of 13.5 log photons/cm^2^/s as measured radiometrically (USB4000 Spectrometer, OceanOptics, Dunedin, FL). Calculated retinal irradiance accounts for age-related lens density and the difference in transmission of red and blue wavelengths through the lens [65]. We will use lights of equal retinal irradiance (13.5 log photons/cm^2^/s) stimuli of 200 ms based on Park et al. [66]. The red and blue exposures will be alternated four times each. Our irradiance is comparable to Gamlin et al. [30] (13.5–14.1 log photons/cm^2^/s) and Zele et al. [67] (14.2 log photons/cm^2^/s). Pre-processing via MATLAB (Natick, MA) for the PIPR includes [1] blink removal, [2] averaging the pupil area, and [3] calculating Net PIPR. Linear interpolation is used to define and replace blink data points using co-investigator’s (K.A.R.) laboratory’s standard methodology [31]. The PIPR will be analyzed for 30 seconds, beginning 10s after light OFF, and both blue and red PIPR will be calculated as a percentage of baseline constriction 7 s prior to stimulus onset. We use an 11 min dark adaptation period just prior to testing, and 45 min in dim light prior to that (< 50 lux). We are using 2 min intervals between stimuli to reduce potential cumulative effect of repeated stimuli. We average all four trials for red and blue, regressed on calculated retinal irradiance for each trial, and then calculate a Net PIPR (blue–red). One of the benefits of the Net PIPR is that drug effects are expected to affect the PIPR response to both blue and red light similarly. The main difference between the red and blue PIPRs is that the blue PIPR includes responses due to intrinsically photosensitive retinal ganglion cells (ipRGCs), while the red does not. Subtracting the red response from the blue leads to a Net PIPR value excluding all influences on pupil dilation except the ipRGC effect. Therefore, effects on pupil dilation of caffeine, nicotine, antidepressant medications, alcohol, exercise, diet, or even autonomic regulation, as examples, in SAD will be controlled mathematically with Net PIPR.

##### Wrist actigraphy

Participants will wear the motion-sensitive ActiGraph GT3XP-BLTE (Pensacola, FL) on their non-dominant wrist for 24-h a day for one week. Actigraphs record the number of movements per unit time, and analysis of these data will provide an objective measure of sleep/wake patterns not subject to recall bias like self-report. Wake time, based on actigraphy, will be entered as a covariate in analyses with the PIPR to control for time since wake and diurnal variation.

##### Pittsburgh Sleep Diary (PghSD)

Concurrent with the actigraphy for one week, participants will complete the PghSD [54], a diary of sleep-wake behavior, via a secured internet website that tracks time of completion to ascertain that the data is truly prospectively collected, rather than sporadically and retrospectively completed. Upon awakening in the morning, questions are as follows: the times at which the participant went to bed and attempted to fall asleep; the number, cause, and duration of nocturnal awakenings; final time out of bed; and total estimated time asleep. Visual analog scales estimate overall sleep quality and mood and alertness upon awakening.

##### Dim light melatonin onset (DLMO)

Estimation of the evening time of DLMO will be conducted at home, following the 1-week actigraphy assessment using salivettes to collect salivary melatonin levels every 30 m from 7 h prior to habitual bedtime until 1 h past habitual bedtime while under dim (< 10 lux) light to prevent acute melatonin suppression by light. Participants will be fitted with dark-colored goggles for 95% light filtration (Uvex Flex Seal with Shade 5.0 Infra Dura lens) to wear for the collection procedure.

##### Phase angle difference (PAD)

PAD will be calculated by determining the number of hours between DLMO and average sleep mid-point (clock time of sleep onset–final morning awakening/2) over actigraphy.

##### EEG/pupillometry

Responses to emotional and seasonal words using a word-valence identification task [38] will be measured using pupil dilation (via infrared pupillometry as described for the PIPR) and frontal gamma-band EEG responses. Stimuli will include emotionally salient words (positive, negative, and neutrally valenced words; 20 each) and seasonal words (winter and summer words; 20 each) presented using E-prime (Psychology Software Tools, Pittsburgh, PA). Word corpi were generated by selecting words with minimal semantic distance from seed words in the GoogleNews corpus (described at https://code.google.com/archive/p/word2vec/#Pre-trained_word_and_phrase_vectors) balanced for word length, frequency, and predicted valence and arousal. The corpus contains approximately 1 billion words, from which 3 million words and phrases have been subjected to machine learning to derive 300 features which, together, predict the probability that any term will occur within a small proximity of another term in the corpus. This technique has been shown to effectively code the words for content [68] using the cosine between the feature vectors as a contextual similarity metric. Valence and arousal predictions were generated based on training a feature classier on normative word ratings from the affective norms for English words (ANEW) [69] word set using seed words for negative (“negative” and “sad”), neutral (“neutral,” “calm,” “unemotional”), positive (“positive,” “happy”), summer (“summer,” “summertime,” “light”), and winter (“winter,” “wintertime,” “dark”). The task design will be largely as we have used in the past [38], though with words presented out loud using text-to-speech recordings to avoid light-reflex responses, following a fixation cross, using an inter-stimulus interval of 8 seconds. The stimulus word lists are displayed in Table 2.

**Table 2:** Stimulus words in the word-valence identification task

Stimulus category				
Seasonal words		Emotionally salient words		
Winter	Summer	Negative	Positive	Neutral
Blizzard Chill Cloudy Cold Damp Darkness December Dreary February Frost Gray Hibernate Icy Indoors Sledding Snow Snowshoeing Snowy Windy Winter	August Autumn Boating Brightest Camp Getaways Heat Hot July June May Midsummer Outdoors Picnics Spring Summer Sunny Sunshine Vacation Warm	Awful Bad Biased Bothers Disgusting Frustrating Hateful Horrendous Horrible Horrid Hostile Negative Shameful Sorry Terrible Ugly Unhelpful Unpleasant Weird Worrying	Cheerful Confident Constructive Delighted Exciting Fantastic Fortunate Grateful Happy Healthy Pleased Satisfied Successful Supportive Terrific Thankful Thrilled Upbeat Uplifting Welcoming	Agnostic Analyst Cautious Dull Flat Hold Impartial Intact Lowered Mediate Preferred Purely Rating Sell Sided Subjective Tact Tense Unbiased Upgraded

Task data will be conducted during a restricted daytime window of assessment, and time of testing as well as individual chronotype or DLMO values will be explored as covariates in analyses to compensate for daily variations in positive affect as well as cognitive influences such as reaction time.

Pupillary reactivity will be collected using the same hardware described for the PIPR, with preprocessing (linear interpolation through artifacts and blinks, averaging of pupillary waveforms across trials within conditions) as in Siegle et al. [70] with mean pupillary reactivity during trials minus a pre-stimulus baseline being evaluated per participant as a dependent measure.

EEG will be measured with BioSemi (Cortech Solutions, Wilmington, NC) ActiveTwo amplifiers at 32 sites using a sampling rate of 250 Hz (1 samp/4 ms) and a .02–100 Hz band-pass filter, subjected to average referencing, artifactual channel interpolation and continuous Morlet wavelet transformation with mean signal, per electrode, in the gamma band region (30–45 hz) extracted as in Kerr et al. [71]. Frontal gamma will be defined as the mean signal across all collected frontal electrodes (Fp1, AF3, F7, F3, FC1, FC5, FC6, FC2, F4, F8, AF4, Fp2, Fz) minus a pre-stimulus (fixation) baseline of 100 ms with secondary analyses [1] separating left and right frontal gamma measures and [2] examining sample-wise tests along extracted pupillary and EEG frontal gamma waveforms to identify temporal intervals of group and condition-related variation, controlling type I error using temporal contiguity thresholding, as in Siegle et al. [70].

##### Self-report measures of the treatment targets and mechanisms

This study uses PIPR, pupil dilation, and EEG because they are relatively inexpensive, and clinical settings could feasibly gain access to a pupilometer or EEG equipment to assess whether the intervention engages associated change mechanisms. However, questionnaire measures tapping similar constructs such as chronotype/circadian entrainment, seasonal beliefs, and depressogenic core beliefs will be included in the pre-, mid-, and post-treatment and follow-up assessment battery and assessed for concordance with the biomarker measures: Composite Scale of Morningness [57], Seasonal Beliefs Questionnaire [32], and the Dysfunctional Attitudes Scale-Form A [55]. Behavioral activation is explored as another potential target and mechanism of CBT-SAD using the Behavioral Activation for Depression Scale (BADS) [56].

##### Winter 2 and 3 follow-up procedures

Follow-up assessments will occur in January of the next two winters (in winters 2 and 3). Testing in January reflects when the vast majority of SAD patients are in episode and when there are sufficient weeks left in the winter to treat them with the alternate modality if we detect a recurrence (i.e., 100% of SAD patients had an annual pattern of depression onset between September and January, 0% remitted before January, and only 22% remitted in January or February) [1]. Consistent with intent-to-treat principles, we will attempt to collect data on all enrolled subjects, including dropouts and those withdrawn from protocol. Participants will be interviewed by a clinical graduate student blind rater with the SIGH-SAD and the SCID-5-RV mood disorders module to assess for a major depressive episode in the interval from treatment endpoint to winter 2 and from winter 2 to winter 3. Participants will be assessed on biomarkers, questionnaire outcomes (BDI-II, BAI, Q-LES-Q, SDS, and MOS-SF-20), and the questionnaires we used in our prior studies to assess treatment utilization in the interim.

### Plans to promote participant retention and complete follow-up {18b}

We will attempt to follow all enrolled participants, including participants who do not complete the treatment or were withdrawn from protocol, consistent with the intent-to-treat principal. This strategy should reduce the frequency of missing data at all time points. In addition, we will implement the tracking and retention strategies that were successfully implemented in the P.I.’s previously completed R01 study where we had very little missing data.

Enrollment will be monitored in weekly team meetings at P.I.’s laboratory, starting the first week of September and continuing each fall/winter season until that year’s recruitment cycle is complete. We will review the number of enrolled participants (overall and the breakdown by sex and race/ethnicity), the number of individuals scheduled for screening in the next week, and the number of individuals who consented but did not qualify based on screening. The project coordinator will maintain a database of our enrolled participants to track the scheduled next study-related visit and the date. Participants who miss an assessment will be called immediately to find out the reason, attempt to reschedule, and encourage their continued participation.

We will attempt to contact subjects lost to follow-up and find out what the barriers are in order to develop ways to address them. Each qualified participant will routinely provide his/her permanent address; all active phone numbers (cell, home, work); email address; and the names and contact information for three people who will know their address and phone number should they move. To maximize sample retention over the interim between study treatment completion in winter 1 and the winter 2 follow-up and between the winter 2 and 3 follow-ups, we will mail each participant a “Stay in Contact” postcard in the fall, a generic holiday card in December, a “Happy Birthday” card on their birthday, and a study newsletter in the spring. The study newsletter will summarize the study without discussing any findings so as not to contaminate subsequent data collection. The purpose of these mailings is two-fold: [1] to maintain rapport and contact with participants as is critical for follow-up evaluations and [2] to maintain current information on participants’ addresses, phone numbers, and email addresses. Participants will be instructed to return the Stay in Contact card with any new contact information. If any mailing is returned unopened, we will attempt to reach the participant by email and phone to verify his/her current address. If we make contact, we will record the correct address in our database. If we cannot reach the participant, we will try to reach the participant by contacting the three people identified by the participant. If we cannot reach a participant through their contact persons, we will use directory assistance and the internet to attempt to locate them.

### Data management {19}

Information about eligibility screening, recruitment, and enrollment will be recorded in an Excel spreadsheet by the Project Coordinator. A separate spreadsheet will be maintained for enrolled subjects and will include subject identification numbers, assigned when a subject meets all eligibility criteria. This spreadsheet will be used to keep track of data collection throughout a subject’s participation in the study, including the dates measures are administered at baseline, during treatment, and at follow-ups. Study personnel will complete all study forms, except those designated for completion by participants. The research assistants will be designated as reviewers of all completed forms for each participant (many of which they will administer anyway) to verify accuracy and completeness before data entry.

The project coordinator will be responsible for data entry, and a graduate student research assistant will be assigned as a second data enterer to circumvent problems related to double data entry by the same person. All data will be independently double-keyed (once by the project coordinator and a second time by the Graduate Student Research Assistant), electronically compared by Co-I/biostatistician (P.M.V.), and reconciled for any discrepancies. No identifiers except subject number will be included in the database files, which will be transferred electronically between the project coordinator and project statistician using encryption software. Each data collection instrument will be entered into a separate data file and will include a sequence number reflecting the measurement schedule, in addition to the date the instrument was administered, to facilitate data linkage and identification of missed measurements. Data will be read into SAS data files and screened for missing, invalid, or illogical values. A written report of questionable data will be sent to clinical study staff for resolution and any unresolved errors will be re-coded as missing. The report will also include a summary of the data received for each subject, so that study staff can check this against their data collection spreadsheet to verify that all data was transferred, entered, and processed.

Individual data files will be named to reflect the data collection instrument and version, so that corrected and updated data files can be compared to earlier versions to verify corrections and numbers of subjects. Backup copies of all files will be written to CD at the time of their creation. Data files will be linked for statistical analyses by subject identification number and measurement schedule sequence number. Treatment group assignment will be identified by linkage to a file associating this information to subject identification number, which will be generated at the time of enrollment. A summary of each subject’s treatment condition will be sent to study staff for confirmation. Data files will regularly be backed up on our University network, where our lab group has a protected-access folder.

It will be important to carefully separate data collection done by observers unmasked to treatment assignment from that done by observers who have to remain masked. Primarily, this will be accomplished by unmasked observers refraining from discussing individual participant treatment assignments in the routine course of the study, participants agreeing to refrain from discussing their treatment with study personnel other than their interventionist, and by separate storage of treatment assignment in the randomization system files, inaccessible to all but designated study personnel for pre-specified purposes. The assigned study treatment code will not be transferred to the main study database to maintain masking of personnel who will be doing data entry, editing, and coding. The Project Statistician will extract a file of treatment assignments from the randomization system at weekly intervals for both data quality assurance and data monitoring. Files merging treatment assignment with participant data will be kept separate from the main database and will not be accessible by masked personnel. Subjects will be given an anonymous identification number that will not be linked to the randomization assignment until the final data analysis. Our database will contain none of the 18 HIPPA-protected personal health identifiers (e.g., name, address, phone, date of birth, SSN). Because electronic data is completely de-linked from personal identifiers, participant confidentiality is protected in transferring data between the P.I.’s laboratory, the University of Vermont Biometrics Department, and the University of Pittsburgh.

All data will be collected at the University of Vermont, but biomarker data will be processed by the University of Pittsburgh collaborators (co-investigators P.L.F., K.A.R., and G.J.S.) who have extensive expertise in our chosen biomarkers. Drs. Franzen and Roecklein will train the study staff at the Vermont site in the biomarker data collection procedures. Co-I P.L.F. will oversee the development, implementation, and analysis of the EEG and pupillary measurement in Vermont, and contribute expertise in the analysis of the data for the proposed project including blink correction, interpolation, and automated data collection. Dr. Franzen will also supervise the Data Manager at the Pittsburgh site in the management of all actigraphy and sleep diary study data, including the development, maintenance, and documentation of data bases for all sleep diary and actigraphy measures. Co-I K.A.R. will specifically be responsible for overseeing the post-illumination pupil response (PIPR) and dim light melatonin onset (DLMO) data management and processing. Co-I G.J.S. will oversee the development of the emotional reactivity tasks, as well as the implementation and analysis of the EEG measures. Once processed and cleaned, final biomarker data will be transferred, as a de-identified database (coded by subject ID numbers), to the Project Biostatistician (P.M.V.) at the Vermont site) for primary analyses.

Effective March 2020 due to the COVID-19 pandemic, all survey measures were moved online using Qualtrics. Each participant is sent a unique link to input responses, and no identifying information is associated with responses. This also removed the need for data entry (and double-data entry) after this change. SIGH-SAD administration was moved from in-person using paper forms to remote videoconference platform with responses entered in REDCap during the live interview.

### Confidentiality {27}

Original data forms will be kept in locked cabinets in the PI’s laboratory (organized by subject ID numbers) and identifiers will be stored separately. We must collect subjects’ names, addresses, telephone numbers, and email addresses to maintain contact, given that our study has a 2-year longitudinal follow-up interval. Only properly authorized and trained individuals who are directly concerned with the study will have access to individually identifiable private information about human subjects: the PI, the project coordinator, the graduate research assistants, the project statistician, members of the Data Safety and Monitoring Board, and members of the University of Vermont IRB (upon audit as part of annual continuing review).

### Plans for collection, laboratory evaluation, and storage of biological specimens for genetic or molecular analysis in this trial/future use {33}

This trial does not involve collecting biological specimens for storage.

## Statistical methods

### Statistical methods for primary and secondary outcomes {20a}

#### Data analysis

##### Aim 1

Logistic regression will be used to assess the effectiveness of treatment with either CBT-SAD or LT on depressive episode recurrence at winter 2. Covariates will be included in some analyses to assess the effect of treatment on outcomes after adjustment for any ongoing treatment reported at follow-up, such as any new treatment, psychotherapy, light therapy use in the CBT-SAD group, and antidepressant medication as in Rohan et al. [14]. An intent-to-treat analysis will be conducted, using multiple imputation (MI) of missing next winter SIGH-SAD scores. Imputed scores will be used to classify depression recurrence status for individuals who dropped out or were lost to follow-up. Imputed values will be generated using the fully conditional specification (FCS) method to obtain regression estimates based on baseline and post-treatment SIGH-SAD (and component 21-item HAM-D and atypical subscale scores), demographic variables, comorbidity, and other baseline patient characteristics. Imputation will be done separately for the CBT-SAD and LT groups because data from our prior study indicate that the relationships between some of these variables and next winter recurrence differ in the two treatments. Ten data sets with imputed values will be generated and a dichotomous recurrence variable will be computed for each subject based on SIGH-SAD criteria. Logistic regression models will be fitted to each of the ten data sets and their parameter estimates will be combined using MI inference methods [43]. SAS PROC MI and PROC MIANALYZE will be used to carry out the imputation and analysis. The “missing at random” assumption of the MI analysis is valid if the imputation model is predictive of outcome. However, if we have an appreciable amount of missing data at follow-up, we will perform a sensitivity analysis using the pattern-mixture model approach (NMAR option in SAS 13.1) to assess the validity of the MI results.

Similar analyses will be performed to compare winter 3 outcomes in the CBT-SAD and LT subjects who did not have a winter 2 recurrence. Secondary analyses will examine the effects of treatment adherence within each treatment and the effect of therapist and therapy group within the CBT-SAD condition.

##### Aim 2

To examine mechanistic differences between CBT-SAD and LT, mixed effects linear regression with subject as a random effect will be used to model biomarker changes during treatment as a function of time, treatment, and their interaction. We will also model the relationship between biomarker change and change depressive symptoms as a function of treatment by letting the regression coefficient differ between CBT-SAD and LT. This will provide a test of whether potential mediators of effect are treatment-specific. The predictive value of biomarkers at baseline, their pre-post treatment change, and the persistence of the change at follow-up will be examined using logistic regression (for post-treatment remission and winter 2 recurrence) and linear regression (for SIGH-SAD and BDI-II scores) with post-treatment and winter 2 outcomes as dependent variables. Interactions between the biomarkers and treatment will be used to test if the associations with outcomes differ in the CBT-SAD and LT groups. Adherence will be included in ancillary analyses to test its effect on the relationships of treatment with biomarkers and LT or CBT-SAD outcomes. The relationships between biomarkers and outcomes will be validated by examining how well they predict post-treatment and winter 3 outcomes for subjects who experience a recurrence at winter 2 and are switched to the alternative treatment. Data from the cross-over subjects will also be analyzed using mixed linear regression to compare within-person differences in pre-treatment biomarkers and their changes during their initial and subsequent treatments. Treatment, time point (winter 1 or 2), and their interaction will be included to test the effect of treatment sequence on within-person differences in biomarker responses to CBT-SAD and LT.

##### Aim 3

Logistic and linear regressions similar to those for Aim 1 will be used to compare the effect of switching from CBT-SAD➔LT versus LT➔CBT-SAD on winter 3 recurrence and depression scores. Post hoc adjustment for any differences between switch groups in baseline characteristics, treatment response, and biomarker profiles will be done by including them as covariates. Mixed linear regression will be used to longitudinally model depression scores over the three winters, with treatment as a time-varying covariate.

##### Aim 4

We will test mediating effects of candidate biomarkers on change in depressive symptoms using parallel process latent growth curve modeling (LGCM) [72–74]. These tests will focus on treatment outcomes through winter 2 and winter 3 follow-up to elucidate the mechanisms underlying the long-term benefit of CBT-SAD. Analyses will include all available information from multiple biomarker assessments and multiple assessments of depressive symptoms (on the SIGH-SAD and BDI-II), modeling latent intercept and linear slope factors for individual biomarkers and symptoms. Each biomarker will be analyzed in a separate model to avoid undue model complexity. Parallel process LGCM allows for estimation of indirect effect magnitude from treatment assignment, through change in biomarker, to change in depressive symptoms, as well as the direct effect from treatment to change in symptoms. We will calculate established mediation effect size indices from these models such as proportion of total possible effect mediated [75]. LGCM will be conducted using full-information maximum likelihood estimation in Mplus [76].

### Interim analyses {21b}

This clinical trial involves low risk interventions that are unlikely to cause excessive adverse events. In fact, an effective treatment for SAD would reduce participants’ depression severity and, hence, reduce their probability of adverse outcomes. Nevertheless, the P.I. will stop the study if an excessive number of protocol-related adverse events do occur. The study as a whole or any given treatment condition within the study will also be stopped due to the following condition: there is clear evidence of harm or harmful side effects of the treatment. The study will not be stopped due to participant noncompliance or dropouts unless the dropout rate is so high that the effective sample size becomes too small to run meaningful statistical analyses, which is not expected based on our prior trials of these interventions. The data will be analyzed after each annual cycle of assessments is completed, both for safety effects by treatment group and for monitoring for futility.

### Methods for additional analyses (e.g. subgroup analyses) {20b}

#### Sex as a biological variable

We will consider sex and other variables (e.g., age, race, ethnicity, estimated prior number of SAD episodes) as potential covariates in secondary analyses. We will also explore potential sex-related differences in the biomarkers and their response to treatment.

### Methods in analysis to handle protocol non-adherence and any statistical methods to handle missing data {20c}

The data analysis is an intent-to-treat analysis with participants analyzed as randomized. Missing data will be handled through multiple-imputation as described in the analysis plan.

### Plans to give access to the full protocol, participant level-data and statistical code {31c}

This trial will share participant-level data (from participants to consent to sharing their data) with the National Institute of Mental Health Data Archive (NDA) upon completion of the study and publication of the results

## Oversight and monitoring

### Composition of the coordinating center and trial steering committee {5d}

The P.I. (K.J.R.) is highly involved in all monitoring functions as part of the general oversight and scientific leadership of the study at the University of Vermont site and will coordinate the monitoring functions of the other study personnel involved. The P.I., project coordinator, and all research assistants directly involved in data collection meet weekly to discuss day-to-day support for the trial. The project biostatistician (P.M.V.) joins these meetings when data management issues are discussed. University of Pittsburg co-investigators join these meetings for the first few weeks of biomarker data collection each year to monitor the quality of the data and engage in an annual site visit where processed data are reviewed. The P.I. meets weekly with co-investigator T.T.P. during active light therapy to make individual adjustments to the light therapy prescriptions and monitor side effects. The P.I. meets weekly with CBT-SAD co-facilitators during active CBT-SAD to supervise the treatment.

### Composition of the data monitoring committee, its role and reporting structure {21a}

This study has a Data Safety and Monitoring Board (DSMB) in place. The three members of the DSMB are university distinguished professors who all have expertise in intervention research and with adult depression populations: Steven Beach (Distinguished Research Professor, University of Georgia), Bruce Compas (Chaired Professor, Vanderbilt University), and Rex Forehand (University Distinguished Professor, University of Vermont). The DSMB will meet annually to monitor the following information: adverse events, subjects removed from protocol, participant accrual and retention, and women and minority inclusion. In advance of each meeting, the P.I. will compile and disseminate a written report on these items. The meeting will begin with the P.I. present to provide an overview and address any questions and then enter a closed discussion. Subsequent to each meeting, the DSMB will prepare a written report that will go the University of Vermont’s IRB.

### Adverse event reporting and harms {22}

The following will constitute adverse events (AEs): (1) serious side effects to light therapy, (2) mania or hypomania, (3) significant suicidal intent, (4) death and/or hospitalization regardless of the reason, and (5) any unexpected, unusual, or severe adverse event. When an adverse event is identified, the P.I. will file a “Report New Information” (RNI) report, including a description of the adverse event and the action that was taken to address the event, with the University of Vermont IRB and the DSMB members, within 24 h of the time the event becomes known to the study investigators. In addition, any unanticipated problems involving risks to participants will be reported to these channels.

(1) Severe side effects to light therapy

Side effects to light therapy will be closely and regularly monitored by the P.I. and co-investigator T.T.P. during light therapy supervision. Participants are instructed not to wait for an appointment if they experience a distressing side effect, but to call the PI immediately. In these cases, Dr. Postolache will be available for emergency telephone consultation with the P.I. The judgment of Dr. Postolache will determine if a reported side effect constitutes an adverse event based on its severity and reversibility. If we identify an adverse event, the P.I. will send a report to the University’s IRB and to members of the DSMB within 24 h, as described above. The P.I. and Dr. Postolache will take immediate action to treat the adverse event (typically by changing the duration or the timing of light therapy). Light-induced mania or hypomania, a rare, but more serious, side effect to light therapy, will always be considered as an adverse event and reported accordingly.

(2) Mania or hypomania

This study recruits individuals with major depression, recurrent, with seasonal pattern. SAD, however, can manifest in a bipolar-type pattern (major depressive episodes that alternate with manic or hypomanic episodes; bipolar I disorder and bipolar II disorder, respectively). Bipolar disorder is an exclusionary criterion for the proposed study. The phone screening and diagnostic interview (Structured Clinical Interview for DSM-5) should rule out most individuals with bipolar-type SAD from participation. However, due to the low validity of bipolar patients’ self-report and to the possibility of a first manic or hypomanic episode onset, it is possible that some participants may develop a manic or hypomanic episode during the study. A manic or hypomanic episode is also a rare side effect to light therapy, as described above. The P.I. or her staff may learn about a manic or hypomanic episode during regular symptom monitoring with the Structured Interview for the Hamilton Depression Rating Scale Seasonal Affective Disorder Version (SIGH-SAD), through behavioral observations or thought content during the group CBT-SAD sessions, or from participants in reporting side effects to light therapy.

If a participant demonstrates evidence of a manic or hypomanic episode, the P.I. will evaluate this further in collaboration with Adult Psychiatry at the University of Vermont-affiliated medical school, which has agreed to consult in these instances, or, if the episode seems to be a side effect to light therapy, with co-I T.T.P. If we identify a manic or hypomanic episode in a study participant, we will take appropriate action, including referral for additional treatment to address the bipolar symptoms. In the event of mania or hypomania related to light therapy, the P.I. and Dr. Postolache will take appropriate action, which may include (a) changing the dose of the light or (b) recommending that the participant stop using light therapy altogether and refer them for additional treatment to address the bipolar symptoms. The P.I. will report this adverse event to the University’s IRB and the DSMB within 24 h of becoming aware of the adverse event.

(3) Significant suicidal intent

Suicidal intent will be regularly assessed at the ongoing symptom monitoring via standard questions on the SIGH-SAD and the Beck Depression Inventory-Second Edition (BDI-II). If a participant expresses suicidal intent spontaneously during the study or on either of these measures, the P.I. will immediately assess the participant further to determine if the participant has suicidal intent significant enough to warrant intervention (e.g., has a plan). If significant suicidal intent is present, the P.I., in collaboration with Adult Psychiatry, will immediately act with appropriate crisis intervention. Significant suicidal intent will be considered an adverse event, which the P.I. will report to the University’s IRB and the DSMB within 24 h of becoming aware of it.

(4) Death and/or hospitalization regardless of the reason.

(5) Any unexpected, unusual, or severe adverse event.

### Frequency and plans for auditing trial conduct {23}

The study will undergo annual continuing review by the University of Vermont IRB.

### Plans for communicating important protocol amendments to relevant parties (e.g., trial participants, ethical committees) {25}

Protocol amendments are formally requested through the University of Vermont IRB. If amendments affect the risk/benefit of participating, current trial participants are informed of the new information in writing.

### Dissemination plans {31a}

The study team intends to publish the results for the study aims. Participant-level data (from those who consent to data sharing) will be shared with the National Institute of Mental Health Data Archive (NDA). The P.I. will communicate the main study findings to study participants in a newsletter after data collection is complete and data have been analyzed.

## Discussion

The COVID-19 pandemic caused significant disruptions in data collection. The University of Vermont went fully remote in March 2020, when we still had 26 participants in active treatment that winter. Although we were able to move clinical interviews (SIGH-SAD) and CBT-SAD to HIPPA-compliant videoconference and surveys to Qualtrics and continue with active light therapy (as participants do it in their homes), the University IRB halted all research procedures not of direct benefit to participants, including our in-lab biomarker assessments.

## Trial status

At the time of this report, we are using protocol version #20, dated January 31, 2022. Recruitment began in September 2018 and will conclude in February 2022.

## Abbreviations

CBT-SAD: SAD-tailored group cognitive-behavioral therapy
ITT: Intent-to-treat
LT: Light therapy
PAD: Phase angle difference
NIMH: National Institute of Mental Health
PIPR: Post-illumination pupil response
RDoC: Research Domain Criteria
SAD: Winter seasonal affective disorder
