# Correction: Elucidating treatment targets and mediators within a confirmatory efficacy trial: study protocol for a randomized controlled trial of cognitive-behavioral therapy vs. light therapy for winter depression

**DOI:** 10.1186/s13063-025-09290-y

**Published:** 2025-11-19

**Authors:** Kelly J. Rohan, Peter L. Franzen, Kathryn A. Roecklein, Greg J. Siegle, David J. Kolko, Teodor T. Postolache, Pamela M. Vacek

**Affiliations:** 1 Department of Psychological Science, University of Vermont, 2 Colchester Avenue, Burlington, VT 05405-0134 USA; 2 Department of Psychiatry, University of Pittsburgh, Thomas Detre Hall, 3811 O’Hara Street, Pittsburgh, PA 15213 USA; 3 Department of Psychology, University of Pittsburgh, 4110 Sennott Square, 210 S Bouquet Street, Pittsburgh, PA 15260 USA; 4 University of Maryland School of Medicine, 655 West Baltimore Street, Baltimore, MD 21201-1559 USA; 5 Biomedical Statistics Research Core, University of Vermont Larner College of Medicine, 25 Hills Building, 111 Colchester Avenue, Burlington, VT 05401-0134 USA

**Correction: Trials 23, 383 (2022)**

**https://doi.org/10.1186/s13063-022-06330-9**

Following the publication of the original article [1], we were notified that their author’s last name was misspelled as Roeckelin instead of Roecklein.

The original article has been corrected.
